# Türkiye’s position in socio-economic inequalities in adult obesity: a gender-specific and regional assessment

**DOI:** 10.1017/S1368980026102031

**Published:** 2026-03-09

**Authors:** Atalay Aktuna, Isil Ergin, Merve Akbayrak, Hur Hassoy

**Affiliations:** 1 Department of Public Health, Bandirma Onyedi Eylul University, Faculty of Medicine, Balikesir, Türkiye; 2 Department of Public Health, Ege University, Faculty of Medicine, Izmir, Türkiye

**Keywords:** Socio-economic inequalities, Obesity, Education, Income, Regional comparison

## Abstract

**Objective:**

This study aims to determine the current status of the obesity epidemic in Türkiye from a global perspective by examining gender-specific socio-economic inequalities at national and regional socio-economic development (SED) levels.

**Design:**

A cross-sectional analysis was conducted using data from the 2022 Türkiye Health Survey, employing weighted binary logistic regression models, age-standardised prevalence estimates for national obesity prevalence and model-based age-adjusted prevalence estimates for regional comparisons, with analyses stratified by sex.

**Setting:**

Türkiye.

**Participants:**

Data included 20 725 nationally representative adults aged 20 years and older (10 808 women and 9917 men).

**Results:**

The national age-standardised obesity prevalence was substantially higher (OR: 1·558; 95 % CI: 1·556, 1·560) in women (28·0 %) than men (18·4 %). In low-SED regions, the gender disparity (women 28·4 %, men 17·9 %) was larger. Higher education was consistently associated with lower obesity risk, more pronounced in women and low-SED regions. The income–obesity relationship was complex. An inverted U-shaped pattern across income quintiles was observed among men in high regional SED and among women both nationwide and across all levels of regional SED.

**Conclusions:**

As of 2022, Türkiye maintains a high obesity prevalence reflecting socio-economic patterns typical of developing countries experiencing nutritional transition. The epidemic stage varies by regional SED, emphasising the necessity for prevention strategies designed with a focus on socio-economic determinants, regional and gender sensitivity.

Obesity, a chronic and complex disease characterised by excess fat accumulation, continues to be defined as a global epidemic that can negatively impact health and remains a leading public health concern^(1)^. In terms of global disease burden, it is estimated that in 2021, a BMI above the optimal level contributed to 3·7 million deaths from chronic diseases worldwide. Obesity is considered one of the major risk factors for noncommunicable diseases. As of 2022, approximately 2·5 billion adults are estimated to be overweight, of which approximately 890 million are classified as obese^(1,2)^.

Although the prevalence and severity of obesity and its adverse health effects are common across all populations, there are marked disparities across different socio-economic groups^(3)^. Moreover, obesity-related health inequalities follow multidimensional patterns that vary according to countries’ levels of economic development and cultural norms. In low- and middle-income countries, the Global South being one evident example, obesity tends to be concentrated among wealthier and more educated populations^(4)^. However, as economies grow, a reversal occurs, with risk shifting from higher to lower socio-economic groups^(4–6)^. The effects of this transition are often apparent earlier in women than in men, resulting in distinctive gender inequalities^(3,4)^. Moreover, even in Northern European countries characterised by relatively high levels of income equality and social welfare, obesity remains consistently higher among lower socio-economic groups, a phenomenon referred to as the ‘Nordic paradox’^(7)^.

According to the WHO European Regional Obesity Report 2022, Türkiye has the highest adult obesity prevalence among fifty-three countries in the region, at 32·1 %^(8)^. Similarly, in the report published by the OECD in 2019, Türkiye ranked third after the United States and Saudi Arabia in both overall obesity and morbid obesity prevalence among OECD accession and partner countries^(9)^. Despite this significant obesity burden, data on socio-economic inequalities in obesity in Türkiye remain scarce and fragmented. A study from nearly two decades ago based on the 2002 World Health Survey revealed region- and gender-specific inequalities. Except for the less developed eastern region, obesity risk was found to be particularly high among middle-income and low-educated women and high-income men. This study also provided evidence that regions within the same country may be at different stages of the obesity epidemic depending on their levels of socio-economic development^(10)^. Although some more recent studies focusing on specific regions^(11)^, genders^(12)^ or social determinants such as income^(13)^ provide valuable information, they do not offer an up-to-date, nationally representative picture of gender-specific socio-economic inequalities in obesity that considers regional comparisons. Therefore, there is still a need to assess potential changes in the national pattern comprehensively and better understand where Türkiye currently stands in the obesity epidemic, especially following the COVID-19 pandemic and ongoing macroeconomic fluctuations. The present study addresses this gap by providing an up-to-date, nationally representative analysis of gender-specific socio-economic inequalities in obesity by education and income across regional socio-economic development strata in Türkiye.

In this context, this study aims to determine the current stage of the obesity epidemic in Türkiye, focusing on gender-specific socio-economic inequalities and their variation by regional socio-economic development.

## Methods

### Data

**Figure 1.**
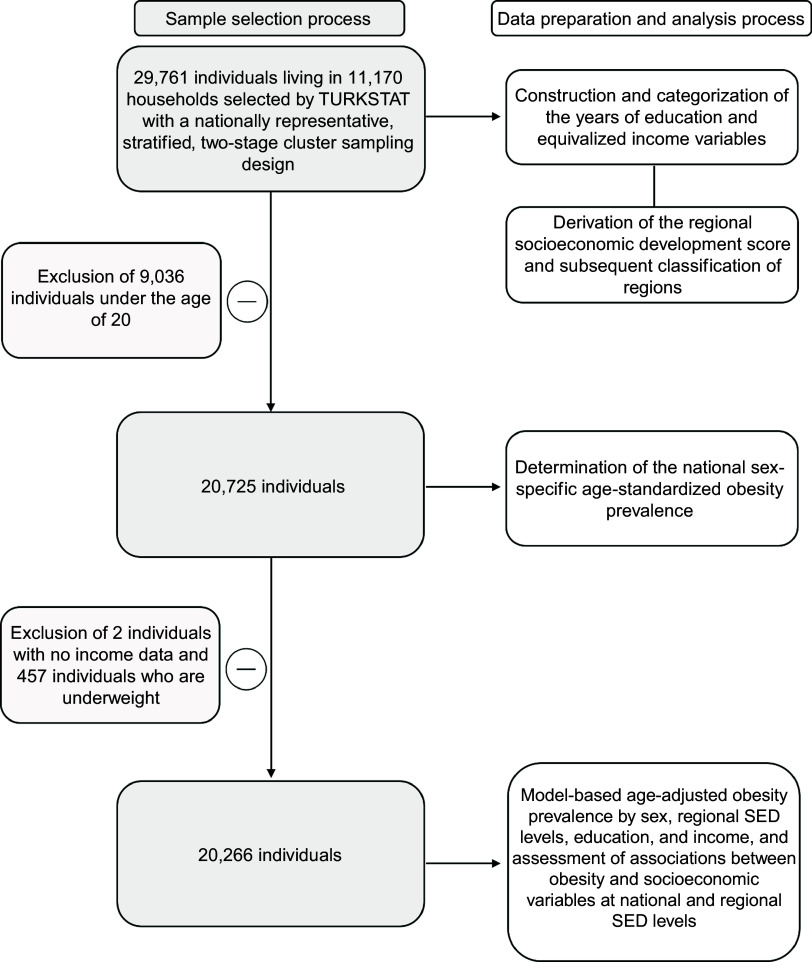
Sample selection, data preparation and analysis processes of the study.

In this cross-sectional study, data from the 2022 Türkiye Health Survey (THS 2022) conducted by the Turkish Statistical Institute (TurkStat) was used. The survey was based on face-to-face interviews with 29 761 individuals living in 11 170 households, selected through a nationally representative, stratified, two-stage cluster sampling design across twelve regions^(14)^. In this secondary data analysis, individuals under the age of 20 (9036 people) were excluded and age-standardised prevalences of obesity were calculated (*n* = 20 725). Underweight individuals (457 people) who were predicted to have a confounding effect in terms of socio-economic determinants^(15)^, and those with missing income data (two people) were excluded from the analysis examining the association of obesity with education and income (*n* = 20 266). The exclusion criteria for anthropometric measurements proposed by the Oxford cohort of the European Prospective Investigation into Cancer and Nutrition (EPIC-Oxford) were reviewed. Regarding these criteria, none of the participants were excluded (e.g. implausible height or weight values)^(16)^. Figure 1 summarises the sample selection, data preparation and analysis process steps detailed above.

### Variables

The dependent variable in this study was obesity. Independent variables included age, sex, regional socio-economic development (SED), education and income. Age was treated as a continuous variable, and analyses were stratified by sex (men/women) and regional SED levels (high/low).

BMI was used to determine obesity. It was calculated using self-reported height (cm) and weight (kg) data in THS 2022 with the standard formula: weight (kg)/height^2^ (m^2^). Based on BMI values, participants were classified as underweight (< 18·50 kg/m^2^), normal weight (18·50–24·99 kg/m^2^), overweight (25·00–29·99 kg/m^2^) or obese (≥ 30·00 kg/m^2^)^(17)^.

Education level was assessed based on the highest level of completed schooling. Participants were categorised into two groups according to years of formal education in Türkiye: less than 8 years and 8 years or more.

In THS 2022, monthly income was evaluated by the household head using a twenty-point ordinal scale ranging from 1, representing income between 0 and 1646 Turkish Lira (TL) to 20, representing income of 14 813 TL and above. To transform this household-level information into an individual-level measure suitable for analysis, we first assigned a continuous income value to each ordinal category using the pre-exclusion sample of 11 170 households. For each category, the median of the income interval was taken as the household income; for the top open-ended category (income of 14 813 TL and above), a value equal to 25 % above the lower bound was used. Second, equivalised household income was calculated using the OECD-modified equivalence scale (weight 1·0 for the first adult, 0·5 for each additional household member aged ≥ 14 years and 0·3 for each child aged < 14 years)^(18)^. The household equivalence size was obtained by summing these weights, and the assigned household income (in TL) was divided by the household equivalence size to derive individual-level equivalised income. Finally, participants were divided into five groups according to equivalised income quintiles: lowest, second lowest, middle, second highest and highest.

Although THS 2022 defines twelve regions, TurkStat does not disclose their names and instead provides only numeric codes, owing to potential limitations in regional representativeness^(14)^. In line with these constraints and the aims of the study, and irrespective of the regions’ actual NUTS-1 identities or official development rankings, we classified the twelve survey regions into socio-economic development categories based on within-survey indicators: years of education and the equivalised income calculated from pre-exclusion data. To categorise the regions according to their level of socio-economic development, a scoring method derived from the Human Development Index (HDI) calculation procedure was applied^(19)^. First, the min-max normalisation method was used to produce standard scores for both education (years) and income (equivalised value) for each region, as shown in Equation (1). Second, the geometric mean of the education and income scores was calculated to obtain an overall regional socio-economic development score ranging from 0 to 1. Based on these composite scores, regions were classified into two socio-economic development groups: six regions with scores above 0·5 (high-SED) and six regions with scores below 0·5 (low-SED). The data used in the equation, including education, income, the composite socio-economic development scores and the resulting classification, are presented in online supplementary material, Supplemental Table 1 by the original region codes.
Eq 1.

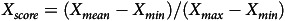




*X*_*score*_: The min-max normalised (0–1) education or income score for a given region.

*X*_*mean*_: The region’s mean education (years) or mean equivalised income.

*X*_*min*_: The minimum of the regional means across all regions.

*X*_*max*_: The maximum of the regional means across all regions.

### Statistical analysis

The national age-standardised obesity prevalence estimates are presented with 95 % CI and were calculated using direct standardisation method based on the 2013 European Standard Population (ESP) in five-year age groups^(20)^. Associations between sex, regional SED, education, income and obesity were examined with age-adjusted binary logistic regression models. Age was included as a continuous variable in all models. Sex, regional SED, education and income were entered as categorical exposure variables, with women, low-SED regions, < 8 years of education and the lowest income quintile serving as the reference categories in models. Separate models were fitted for each exposure (sex, regional SED, education or income), rather than including these exposures simultaneously in the same model, in order to avoid potential over-adjustment when modelling correlated socio-economic indicators and to facilitate interpretation of each gradient. Model-based age-adjusted prevalence estimates were obtained from binary logistic regression models by predicting the probability of obesity for each individual and averaging these predicted probabilities within the relevant subgroups. All analyses were weighted using the individual sampling weight (FERTFAKTOR) provided by TurkStat, which represents the inflation factor for each interviewed person^(14)^. Data analyses were performed using SPSS (version 24; IBM Corporation), and visualisations were created using Microsoft Excel. A *P* value of < 0·05 was considered statistically significant.

## Results

The distribution of the surveyed population aged 20 and over according to sex, socio-economic indicators and SED levels of regions are presented in Table 1. Among the surveyed population aged 20 years and over, 50·8 % were women. The mean (sd) of ages was 45·8 (sd 16·8) years for women and 44·7 (sd 16·0) years for men. In terms of educational attainment, 51·1 % of women and 35·7 % of men had less than 8 years of education. Furthermore, 53·8 % of men and 57·4 % of women belonged to income levels middle or below. The proportions residing in regions with high and low-SED regions were 62·7 % (men: 63·1; women: 62·3) and 37·3 % (men: 36·9; women: 37·7), respectively. Descriptive data excluding underweight individuals and those with missing income data are presented in online supplementary material, Supplemental Table 2.

**Table 1.** Numbers (*n*) and distribution (%) of the surveyed population according to sex, socio-economic indicators and regional development

Regional development	High		Low		Total	
	Unweighted/Weighted		Unweighted/Weighted		Unweighted/Weighted	
	*n*	%	*n*	%	*n*	%
Men						
**Education						
** < 8 years	2133/6 020 629	33·1	1450/4 261 492	40·2	3583/10 282 121	35·7
** ≥ 8 years	4226/12 162 972	66·9	2108/6 351 959	59·8	6334/18 514 931	64·3
**Income						
** Highest	1975/5 476 964	30·1	568/1 551 618	14·6	2543/7 028 581	24·4
** Second highest	1574/4 605 149	25·3	574/1 669 502	15·7	2148/6 274 650	21·8
** Middle	1356/3 791 440	20·9	678/1 912 950	18·0	2034/5 704 390	19·8
** Second lowest	1023/2 851 132	15·7	789/2 233 758	21·0	1812/5 084 890	17·7
** Lowest	431/1 458 917	8·0	949/3 245 623	30·6	1380/4 704 540	16·3
Women						
**Education						
** < 8 years	3143/8 633 052	46·6	2323/6 556 811	58·4	5466/15 189 863	51·1
** ≥ 8 years	3692/9 896 840	53·4	1650/4 667 413	41·6	5342/14 564 253	48·9
**Income						
** Highest	1952/5 008 688	27·0	570/1 439 362	12·8	2522/6 448 050	21·7
** Second highest	1623/4 448 987	24·0	637/1 762 468	15·7	2260/6 211 455	20·9
** Middle	1585/4 322 995	23·3	811/2 217 318	19·8	2396/6 540 312	22·0
** Second lowest	1137/3 068 108	16·6	894/2 409 510	21·5	2031/5 477 618	18·4
** Lowest	536/1 676 364	9·0	1061/3 395 567	30·3	1597/5 071 930	17·0

**Figure 2.**
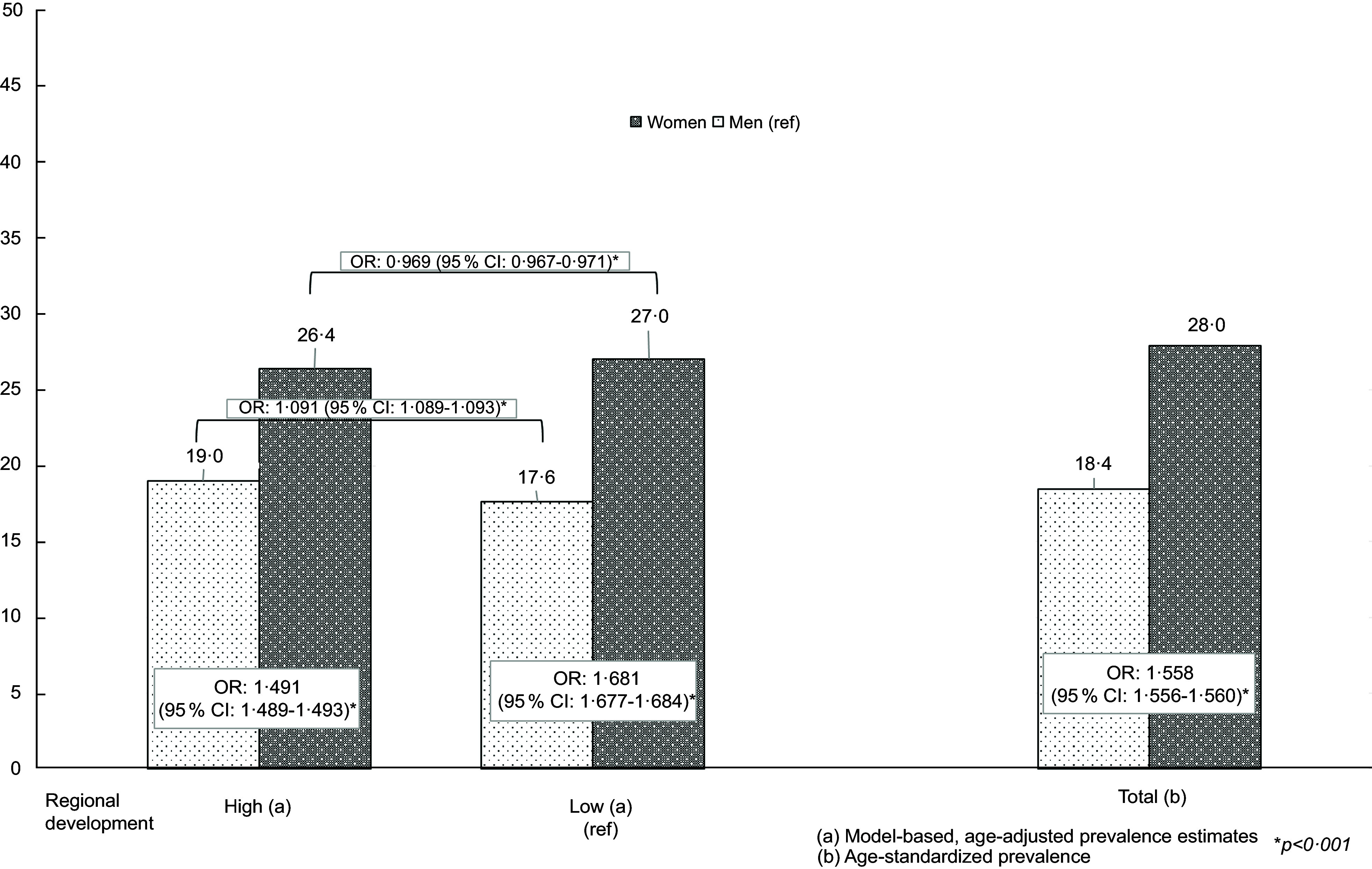
Sex-specific obesity prevalence estimates (%) and OR by regional socio-economic development.

The national age-standardised obesity prevalence rates among adults in Türkiye for 2022 were 27·96 % (95 % CI: 27·94, 27·98) in women and 18·45 % (95 % CI: 18·43, 18·46) in men, indicating a 1·56-fold (OR: 1·558; 95 % CI: 1·556, 1·560) higher prevalence among women (Δ = 9·5 %). At the regional level, obesity prevalence in women was substantially higher than in men in both high-SED regions (OR: 1·491; 95 % CI: 1·489, 1·494) and low-SED regions (OR: 1·681; 95 % CI: 1·677, 1·684), with the gender disparity being more pronounced in low-SED regions. Among men in high-SED regions, obesity prevalence was 19·0 % (95 % CI: 19·002, 19·004) and was significantly higher (OR: 1·091; 95 % CI: 1·089, 1·093) than in low-SED regions (17·6 %; 95 % CI: 17·634, 17·639). Among women, regional variability was greater; obesity prevalence was 26·4 (95 % CI: 26·402, 26·411) and was significantly lower in high-SED regions (OR: 0·969; 95 % CI: 0·967, 0·971) than in low-SED regions (27·0 %; 95 % CI: 27·034, 27·047). Figure 2 shows the age-standardised obesity prevalence rates for both sexes across regional SED levels and nationwide.

**Figure 3.**
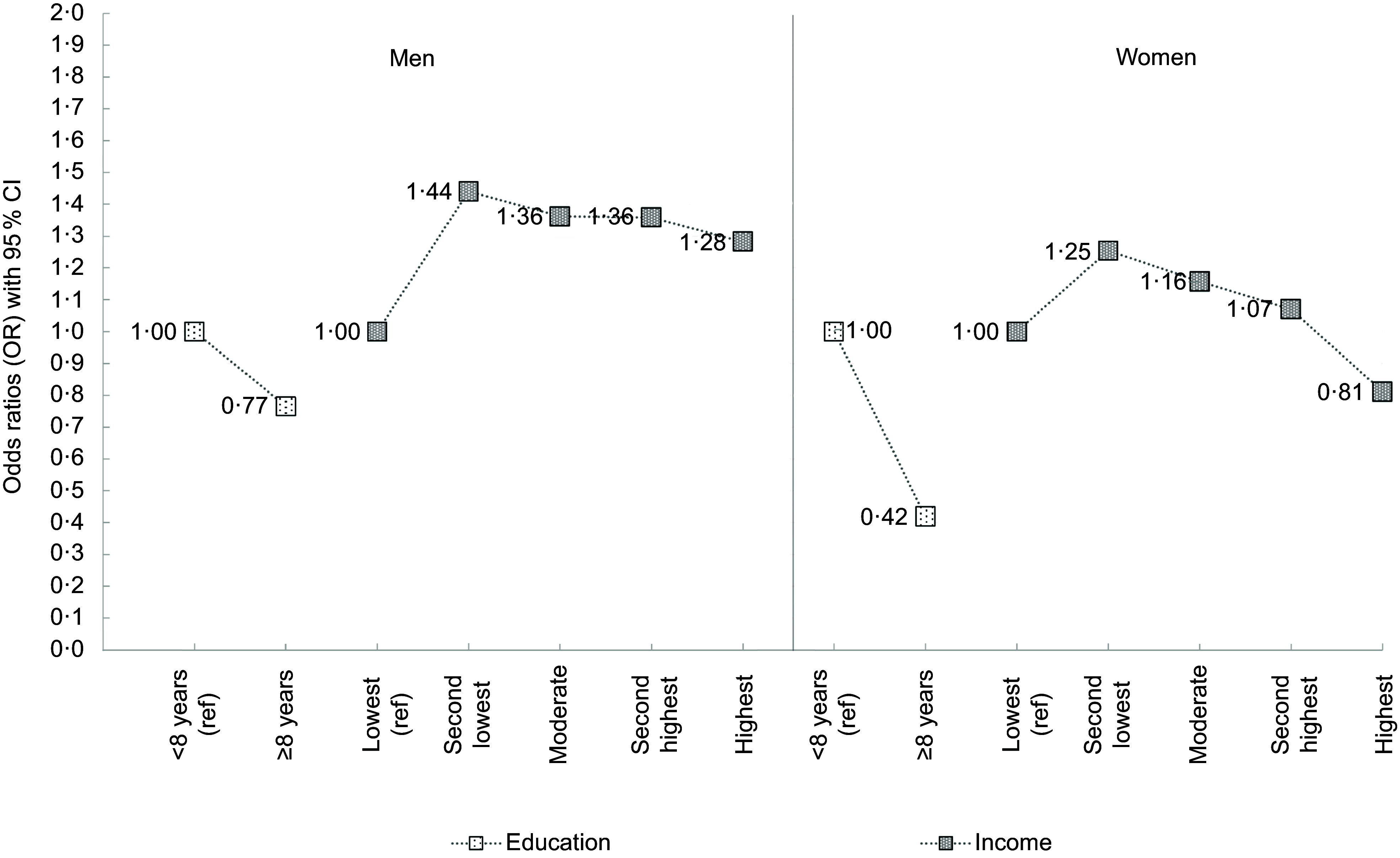
Sex-specific, age-adjusted obesity risk (OR with 95 % CI) according to education and income among Turkish adults.

A high education level (≥ 8 years) was associated with a reduced risk of obesity in men (OR: 0·766; 95 % CI: 0·764, 0·767), and more prominently in women (OR: 0·419; 95 % CI: 0·418, 0·419) across the country (Figure 3). In men, those with high education (≥ 8 years) have a decreased risk for obesity at both regional SED levels, with the association being more pronounced in high-SED regions (OR: 0·668; 95 % CI: 0·666, 0·669). In women, higher educational attainment (≥ 8 years) was similarly associated with lower risk for obesity at both regional SED levels, with comparable OR across SED strata and more pronounced than in men. Table 2 summarises the prevalence rates (%) for obesity and OR with 95 % CI according to educational attainment.

**Table 2.** Model-Based, age-adjusted prevalence estimates (95 % CI) for obesity and odds ratios (OR) by regional development, education and income among Turkish adults

Regional development	Socio-economic indicator	Men				Women			
		Prevalence, %	95 % CI	OR	95 % CI	Prevalence, %	95 % CI	OR	95 % CI
High	Education								
	≥ 8 years	16·1	16·1, 16·1	0·67	0·67, 0·67^*^	15·1	15·6, 15·6	0·40	0·40, 0·40^*^
	< 8 years (ref)	24·9	24·9, 24·9	1·00		38·7	38·2, 38·2	1·00	
	Income								
	Highest	17·5	17·5, 17·5	1·02	1·02, 1·03^*^	20·8	20·8, 20·8	0·90	0·89, 0·90^*^
	Second highest	18·6	18·6, 18·6	1·06	1·06, 1·07^*^	26·7	26·7, 26·7	1·17	1·16, 1·17^*^
	Middle	20·2	20·2, 20·2	1·17	1·16, 1·17^*^	29·8	29·8, 29·8	1·27	1·27, 1·28^*^
	Second lowest	21·7	21·7, 21·7	1·29	1·28, 1·30^*^	31·5	31·5, 31·5	1·54	1·53, 1·55^*^
	Lowest (ref)	17·5	17·5, 17·5	1·00		24·0	24·0, 24·0	1·00	
Low	Education								
	≥ 8 years	15·6	15·6, 15·6	0·95	0·94, 0·95^*^	14·8	13·8, 13·8	0·45	0·44, 0·45^*^
	< 8 years (ref)	20·7	20·7, 20·7	1·00		35·4	36·1, 36·1	1·00	
	Income								
	Highest	19·2	19·2, 19·2	1·56	1·55, 1·57^*^	20·8	20·8, 20·8	0·80	0·79, 0·80^*^
	Second highest	21·7	21·7, 21·7	1·72	1·71, 1·73^*^	29·0	28·9, 29·0	1·08	1·08, 1·09^*^
	Middle	18·9	18·9, 19·0	1·37	1·37, 1·38^*^	32·0	31·9, 32·0	1·14	1·13, 1·14^*^
	Second lowest	18·7	18·7, 18·7	1·41	1·40, 1·42^*^	27·3	27·3, 27·3	1·06	1·05, 1·06^*^
	Lowest (ref)	13·3	13·3, 13·3	1·00		25·2	25·2, 25·2	1·00	

* *P* < 0·001.

Among women at the national level, compared with the lowest income group, there was an increase in risk in second lowest (OR: 1·254; 95 % CI: 1·250, 1·258), middle (OR: 1·157; 95 % CI: 1·154, 1·160) and second highest (OR: 1·070; 95 % CI: 1·067, 1·073) income groups but decreased in the highest income group (OR: 0·811; 95 % CI: 0·809, 0·814). This increase was greatest in the second lowest income group, yielding an inverted U-shaped pattern across income (Figure 3). A similar pattern was observed in both SED levels, with the peak in risk being in the middle-income group in low-SED regions. A broadly similar pattern was seen at both regional SED levels, although in low-SED regions, the peak in risk occurred in the middle-income group. Among men, nationally and at both regional SED levels, all higher income groups had increased risk of obesity compared with the lowest income group. The greatest increase was observed in the second lowest income group at the national level (OR: 1·440; 95 % CI: 1·435, 1·445) and in high-SED regions (OR: 1·288; 95 % CI: 1·281, 1·295), whereas in low-SED regions, the highest risk was found in the second-highest income group (OR: 1·719; 95 % CI: 1·710, 1·727). Thus, the inverted U-shaped pattern seen among women was evident for men only in high-SED regions. The age-standardised prevalence rates for obesity and OR with 95 % CI according to income level are detailed in Table 2.

## Discussion

### Summary of key findings

This study, based on nationally representative data from the 2022 Türkiye Health Survey, investigated gender-specific socio-economic inequalities in obesity among adults at the national level and across regional socio-economic development groups. In the study here in, the age-standardised prevalence of obesity in Türkiye was considerably higher in women (28·0 %) than in men (18·4 %). The gender gap was evident in both high- and low-SED regions but was more pronounced in low-SED regions. While men had slightly higher obesity prevalence in high-SED than in low-SED regions, women showed the opposite pattern and inter-regional variability in prevalence was greater among women. Higher education levels were associated with decreased obesity risk for both sexes at the national level and at both regional SED levels. This association was more pronounced among women and men in high-SED level. For income, among women, compared with the lowest income group, obesity risk increased in the second-lowest, middle and second-highest income groups but decreased in the highest income group, yielding an inverted U-shaped pattern nationally. A broadly similar pattern was seen within both SED strata, although in low-SED regions, the peak in risk occurred in the middle-income group. Among men, all higher income groups had an increased risk of obesity compared with the lowest income group at the national level and in both SED strata. An inverted U-shaped pattern was evident only in high-SED regions, whereas in low-SED regions the largest excess risk was observed in the second-highest income group.

### Interpretation of findings

In the study herein, the age-standardised obesity prevalence in Türkiye was 28·0 % for women and 18·4 % for men as of 2022, clearly indicating that the country continues to exhibit a significantly higher obesity burden not only compared with high-income countries but also compared to the global average. According to the latest global estimates by the NCD Risk Factors Collaboration (NCD-RisC), based on a pooled dataset of 3663 nationally representative population-based studies, the global obesity prevalence in 2022 is 18·5 % for women and 14·0 % for men^(15)^. Eurostat’s 2022 data show that the obesity prevalence in the European Union is around 15 % for women and 17 % for men^(21)^. Similarly, the OECD Health at a Glance 2024 report indicates that the adult obesity prevalence among OECD countries is around 14 % for women and 16 % for men^(22)^.

In Türkiye, in line with international literature^(15)^, the prevalence of obesity among women continues to be significantly higher than among men as of 2022. Although gender differences in obesity are often discussed in relation to biological factors^(23)^, numerous studies have demonstrated that socio-economic and sociocultural determinants are still the primary drivers of gender-based obesity inequalities. Especially in middle-income countries, obesity prevalence among women is fundamentally higher than among men^(3,24,25)^. In contrast, in high-income countries, partly due to the biological predisposition of men to obesity^(23)^, this gender gap tends to narrow or even reverse. Indeed, 2022 European data show that in nineteen out of twenty-six countries, obesity prevalence is now higher among men than women^(21)^. Similarly, a study conducted in South Korea by Nam GE *et al.* (2020) reported that since 2005, obesity prevalence has consistently been higher in men than in women^(26)^. Conversely, in a multilevel analysis conducted across eight Latin American countries, the prevalence of obesity among women (24·7 %) was observed to be approximately 1·3 times higher than that among men (18·4 %)^(27)^. A study by Dade *et al.* (2022) in Haiti, a lower-middle income country, it was stated that the obesity prevalence among women was approximately six times higher than among men (26·5 % *v*. 4·3 %), and 89·2 % of obese participants were women. In the same study, it was determined that behavioural factors that frequently cause obesity, such as nutrition, physical activity and smoking, were not associated with obesity in either gender and gender differences were attributed to sociocultural and socio-economic determinants^(28)^. In our study, the findings that women had approximately 1·6 times higher obesity prevalence than men and that a more prominent gender disparity was observed in low-SED regions, aligns with the existing literature. Furthermore, the greater variability in obesity prevalence among women across regional SED levels, with higher prevalence in low-SED than in high-SED regions, supports the concept that socio-economic exposures affect women and men differently, with the impact of these exposures being more readily visible among women in terms of obesity risk^(3,4,25)^.

In general, it is recognised that the social and economic environment can contribute to obesity through diet and physical activity. An obesogenic environment is defined as one that promotes the consumption of high-energy foods and sedentary behaviours^(17)^. According to the 2023 Türkiye Household Health Survey, 12·1 % of the population (12·4 % of women and 11·8 % of men) consumes at least five servings of fruit and/or vegetables per day^(29)^. Similar rates are reported in the Health at a Glance 2023 report for Mediterranean countries such as Greece (12 %), Spain (11 %) and Italy (11 %)^(30)^. On the other hand, it is noteworthy that nutrition surveys in these Mediterranean countries^(31–33)^ have shown similar caloric intake levels between men and women in Türkiye. Despite this similarity, Türkiye ranked as the seventh-highest country globally in 2022 when considering per capita daily calorie intake (3785 kcal), surpassing Italy (3667 kcal), Greece (3389 kcal) and Spain (3356 kcal)^(34)^. While these figures do not directly reflect actual consumption, they represent energy availability and potential consumption capacity within a country and therefore can serve as a more critical indicator of an obesogenic environment^(34)^. Indeed, obesity prevalence rates in these Mediterranean countries are significantly lower than in Türkiye, and unlike Türkiye, they are higher in men than in women. The Health at a Glance 2024 Report also shows that obesity prevalence was 12·1 % for Greek men, 11·5 % for women, 16·1 % for Spanish men, 13·4 % for women and 7·9 % for Italian men and 6·1 % for women in 2022^(22)^. Intercalarily, the same report also shows that Türkiye records the lowest levels of physical activity among the compared countries. While 35 % of adults in Spain and 19 % in both Italy and Greece engage in at least 150 min of physical activity per week, this figure is only around 5 % in Türkiye^(22)^. The 2023 Türkiye Household Health Survey also shows that 25·9 % of men and 38·4 % of women do not meet the WHO recommendations for health-enhancing physical activity^(29)^ and, compared with other countries, gender disparities in physical activity levels are more pronounced in Türkiye^(22,30)^. The insufficient physical activity levels observed among women in Türkiye are primarily shaped by structural factors inherent to gender roles, such as caregiving responsibilities, time constraints and the ‘ethic of care.’ In addition to these, lack of intra-household social support, patriarchal norms and economic limitations further contribute to the problem. These multilayered barriers undermine women’s ability to autonomously use their leisure time, while the limited availability of gender-sensitive and accessible physical activity environments further restricts their participation^(35)^. The combination of unhealthy diets with more calorie-dense foods and low physical activity observed in both sexes contributes to the high prevalence of obesity in the country. However, compared to other Mediterranean countries with similar dietary habits, the more pronounced gender disparity (favouring women) and the higher overall prevalence suggest that the environment driving obesity in Türkiye is insufficient physical activity, rather than diet, and that this effect is particularly pronounced in women.

In our study, the gender gap in obesity was most pronounced in low-SED regions, and the direction of the SED gradient differed by sex: among men, obesity prevalence was higher in high-SED than in low-SED regions, whereas among women it was lower in high-SED than in low-SED regions, with greater regional variability. These patterns are consistent with differences in social and cultural environments that shape diet and physical activity in Türkiye^(10,35–37)^. Analyses of nationally representative household budget data show that Turkish diets remain largely cereal-based, but increases in household expenditure are associated with disproportionate rises in fat and protein intake, and energy and macronutrient consumption differ across income groups and between urban and rural areas^(36)^. In more developed, urbanised settings, higher incomes, service-sector employment, motorised transport and the greater availability and marketing of energy-dense processed foods plausibly create more sedentary, obesogenic lifestyles, which may contribute to the higher obesity prevalence observed among men in high-SED regions^(36,37)^. By contrast, in less developed settings, opportunities for leisure-time physical activity are generally more restricted for women than for men, as patriarchal gender norms limit women’s ability to leave the home for recreational activities and constrain their access to education, paid employment and welfare resources^(10,35–37)^. In these contexts, women’s social lives are more likely to be organised around home-based gatherings that often involve the preparation and consumption of energy-dense foods, whereas men more frequently participate in outdoor and sport-related social activities and tend to consume animal-source foods more regularly^(10,35–37)^. At the same time, fertility and gender-role expectations may shape aesthetic norms and cultural expectations around body weight, with higher fertility, lower female employment and more conservative gender ideologies concentrated in less socio-economically developed areas^(10,35–37)^. Taken together, these gendered constraints on physical activity, diet and social participation provide a plausible explanation for the higher female obesity prevalence and larger gender disparity observed in low-SED regions^(10,35–37)^.

The role of education, determined in previous studies as a social determinant in obesity with lifelong impact, is more pronounced than that of income, consistent with the results in this study^(24,38)^. In low-income countries and those in the early stages of economic development, higher socio-economic status, including higher levels of education, is positively associated with obesity for both men and women^(3)^. As the country’s level of development and income increase, the relationship between education and obesity reverses, and this transformation primarily makes the protective effect of education apparent, especially among women^(4,5)^. One possible explanation may be that the capacity to cope with the obesogenic environment increases with socio-economic development, both at the societal and individual levels^(3,4,24)^. In this study, higher education (≥ 8 years) was associated with a lower risk of obesity in both women and men at the national level and across both regional SED strata, with the association being substantially stronger in women and more evident in high-SED regions among men, in line with the relevant literature. The study conducted in urban areas of Latin America found a negative association between education and obesity in women (i.e. higher education associated with lower obesity), regardless of the socio-economic development level of the city. The same study reported that for men, the protective effect of higher education was observed in more developed cities^(27)^. The noticeable consistency between that study and our findings suggests that Türkiye exhibits a similar pattern to Latin American countries, where the level of income is comparable^(39)^. Moreover, the obesity prevalence of 24·7 % in women and 18·4 % in men in that study^(27)^ is remarkably close to the rates observed in our study (28·0 % for women and 18·4 % for men). Our findings also show that the significant obesity inequalities based on education observed in women in the 2002 data^(10)^ continues herein 2022, while a similar pattern of inequality among men began to become visible as of 2022, especially in more developed regions, unlike 2002. Furthermore, 2022 data from Mediterranean countries such as Italy, Greece and Spain similarly support the existence of education-based obesity inequalities by gender, suggesting the possible persistence of a broader ‘Mediterranean’ pattern^(21)^. In line with this, another study conducted across eleven European countries also found that lower levels of education were associated with higher obesity prevalence^(38)^. Our findings indicate that Türkiye and European countries, especially Mediterranean countries, may be comparable in terms of gender-based obesity inequalities based on education, despite these countries having much lower obesity prevalence compared with Türkiye. Moreover, the UNDP 2023 Human Development Report lists Türkiye’s education inequality indicator as 13·6 %. The corresponding figures are 15·7 % for Spain, 10·1 % for Italy, 11·7 % for Greece, 13·5 % for Mexico, 14·3 % for Peru, 14·6 % for Colombia and 15·7 % for Brazil among Latin American countries^(40)^. These figures, which position Türkiye between the Mediterranean and Latin American profiles in terms of educational inequality, may indicate that educational inequalities have a strong impact on obesity beyond simple geographic proximity.

In this study, the relationship between income level and obesity shows that, at the national level, obesity risk increases in both women and men as income rises compared with the lowest income group. However, this increased risk follows a decreasing gradient from the second quintile to the highest quintile among women, while no clear pattern is observed among men. The increase in obesity risk with increasing income does not show a consistent pattern at the regional level for both genders. Studies from Europe and other high-income countries demonstrate a higher obesity risk among lower-income groups^(38)^. In contrast, in developing countries, the relationship between income and obesity becomes more complex. In developing countries, obesity, which is initially concentrated in higher-income and urban areas, spreads to the general population and especially to low-income groups as economic development and urbanisation progress, which is described as a result of the nutrition transition^(3,5,41)^. The findings of this study suggest that the effects of the nutrition transition persist in 2022. The protective effect of absolute poverty against obesity, as emphasised in the literature, still appears to apply to the lowest income groups in Türkiye. Individuals in these groups are more likely to experience constrained food budgets, food insecurity and physically demanding domestic or informal work, which may limit weight gain despite unfavorable living conditions. Arguably, individuals who emerge from absolute poverty progress one step further in the nutrition transition, facing a higher risk of obesity as a result of increased and more stable household resources, urbanisation and access to cheap, energy-dense processed foods without improvements in physical activity opportunities, health literacy and supportive nutritional environments^(3,5,41)^. This combination of greater exposure to obesogenic environments and persistent structural constraints may help explain why, in our data, the highest obesity risk among women is observed in the second lowest and middle-income groups, resulting in an inverted U-shaped pattern. By contrast, in the highest income group, better access to health information and services, stronger social norms around body weight and greater control over diet and leisure-time activity may contribute to a modest reduction in obesity risk among women^(24,25)^. This pattern is consistent with the ‘obesity Kuznets curve’ hypothesis, which posits a non-linear, inverted U-shaped relationship between economic development and obesity, with obesity rates first increasing and then declining as income rises beyond a certain threshold^(5,42)^. The fact that the increase in obesity risk among men is stronger in higher income groups in low-SED regions may be interpreted as men are following a trajectory of obesity inequalities that has already been observed among women^(5)^. Additionally, Templin *et al.* demonstrated that at the threshold of $8000–$10 000 per capita GDP in countries, the burden of obesity and overweight begins to shift from the wealthiest 20 % to lower income quintiles. This shift accelerates as national income increases, leading to growing risks among middle- and lower-income groups^(6)^. As of 2022, Türkiye’s GDP per capita is $10 675^(43)^, which supports the conclusion that the country remains in the nutrition transition phase identified in this study. Furthermore, while the highest obesity burden is seen in the lowest-income group in Mediterranean countries such as Italy, Spain and Greece, where nutritional behaviours are similar to those of Türkiye, inequality patterns similar to those observed in Türkiye are seen in Latin American countries that are closer to Türkiye in terms of income inequality^(43)^. Recent evidence indicates an obesity gap between the poorest and richest quintiles that disadvantages the poorest by roughly 1·2 % in Italy, 3·6 % in Spain and 2·3 % in Greece^(44)^. By contrast, Jiwani *et al.*’s 2019 analysis across thirteen Latin American and Caribbean nations found that, in many countries, obesity among women has been shifting towards lower-income quintiles, while among men the association is weaker and more heterogeneous^(45)^. Thus, as with education, income appears to shape obesity through mechanisms that extend beyond geographical proximity or shared dietary habits.

### Strengths and limitations

This study is based on the most recent and nationally representative dataset currently available in Türkiye and adds novel evidence on gender-specific socio-economic inequalities in obesity in an upper-middle-income country undergoing a nutrition transition. Furthermore, evidence on how gender-specific socio-economic inequalities in obesity vary across regions at different stages of socio-economic development is still scarce in Türkiye and in similar middle-income settings^(10–13,46)^, which highlights the originality and relevance of the present study. Missing data were negligible. This enhances the generalisability of the findings. However, the study has several data limitations. First, the data are based on self-reporting, which may introduce certain biases in the results. Previous studies have shown that individuals with higher BMI tend to underreport their weight and overreport their height, which may lead to misclassification bias and an underestimation of the prevalence of obesity^(47)^. Moreover, there is evidence that misclassification of obesity based on self-reported BMI is more pronounced in lower-income and lower-educated groups, particularly among women with lower education^(48)^, which is likely to lead to an underestimation of socio-economic inequalities in obesity; if similar patterns exist in Türkiye, our estimates of obesity prevalence and related socio-economic inequalities are therefore likely to be conservative. Second, the assessment of socio-economic indicators was limited to self-reported education and income at the individual level and did not include longer-term measures such as wealth and occupation. This may have influenced the magnitude of the observed associations, as previous studies have reported that under-reporting and non-response among higher-income respondents, together with a tendency to round income upwards at lower income levels^(49)^, can lead to an underestimation of socio-economic inequalities. However, it is not expected to influence the direction of the association of education and income with obesity^(24,38)^. Furthermore, due to the anonymisation of the regions in the data, the interpretation of regional differences in obesity is limited to indirect evidence on regional variations in social norms, gender roles and food environments. On the other hand, regional comparisons are appropriately discussed in terms of socio-economic development levels that are consistent with the objectives and rationale of the study. Finally, the direction of causality is unclear, an inherent limitation of the cross-sectional design.

### Conclusion

By 2022, Türkiye continues to bear both a high prevalence of obesity and the typical characteristics of the transition that obesity inequalities present in developing countries. In particular, our findings position Türkiye closer to Latin American countries with respect to its patterns of socio-economic development and inequality, thereby demonstrating the extent to which socioe-conomic determinants strongly shape the burden of obesity on global scale beyond the influences of geographic proximity or shared dietary behaviors. It was determined that the epidemic may be at different phases in regions with different levels of socio-economic development within the country. The findings once again underscore the need for obesity prevention strategies to adopt regional and gender sensitive approaches and to focus on socio-economic inequalities in strategic goals. Regarding the income inequalities for obesity, previous suggestions^(50)^ such as implementing gradual taxes on unhealthy foods and using the generated tax revenue to subsidise fruits and vegetables could be effective in improving access to healthy foods. Gender-sensitive and community-based physical activity support programmes should be developed and implemented urgently. Women, particularly those with lower levels of education, who already occupy a disadvantaged position across various health outcomes, face an even greater risk of further deepening health inequalities as a result of the disproportionate obesity burden. Therefore, obesity prevention interventions and empowerment efforts should prioritise this group.

## Supporting information

10.1017/S1368980026102031.sm001 Aktuna et al. supplementary material 1 Aktuna et al. supplementary material

10.1017/S1368980026102031.sm002 Aktuna et al. supplementary material 2 Aktuna et al. supplementary material

## Supplementary material

For supplementary material accompanying this paper visit https://doi.org/10.1017/S1368980026102031
