# Supplementary material for: Türkiye’s position in socio-economic inequalities in adult obesity: a gender-specific and regional assessment
Source: Public Health Nutr. 2026 Mar 9;29(1):e83. doi: 10.1017/S1368980026102031 (PMC13411683; doi:10.1017/S1368980026102031)
Supplement: Aktuna et al. supplementary material 1 — Aktuna et al. supplementary material [file S1368980026102031sup001.docx]

**Supplementary Table 1.** Regional years of education and equivalized income, normalized scores, composite socioeconomic development (SED) score, and classification by original region code

| Numeric code of region | Education (years) | | Equivalent income (TL) | | Overall  SED score | SED Classification |
| --- | --- | --- | --- | --- | --- | --- |
|  | Mean | Score | Mean | Score |  |  |
| 9 | 8.83^*^ | 1.00 | 5,452.9^*^ | 1.00 | 1.00 | High |
| 2 | 8.74 | 0.96 | 4,874.3 | 0.79 | 0.87 | High |
| 6 | 8.42 | 0.80 | 5,053.3 | 0.85 | 0.83 | High |
| 7 | 8.33 | 0.76 | 4,941.1 | 0.81 | 0.79 | High |
| 4 | 8.09 | 0.65 | 5,155.1 | 0.89 | 0.76 | High |
| 11 | 8.20 | 0.70 | 4,309.5 | 0.58 | 0.63 | High |
| 1 | 7.77 | 0.49 | 3,812.5 | 0.39 | 0.44 | Low |
| 3 | 7.38 | 0.30 | 4,217.2 | 0.54 | 0.41 | Low |
| 5 | 7.26 | 0.25 | 4,029.4 | 0.47 | 0.34 | Low |
| 12 | 6.99 | 0.12 | 2,857.1 | 0.04 | 0.07 | Low |
| 10 | 6.74^◦^ | 0.00 | 2,866.9 | 0.05 | 0.00 | Low |
| 8 | 6.75 | 0.00 | 2,742.0^◦^ | 0.00 | 0.00 | Low |

^◦^Minimum and ^*^maximum regional means of years of education and equivalent income value used in the relevant equation (Eq 1)
