# Supplementary material for: Türkiye’s position in socio-economic inequalities in adult obesity: a gender-specific and regional assessment
Source: Public Health Nutr. 2026 Mar 9;29(1):e83. doi: 10.1017/S1368980026102031 (PMC13411683; doi:10.1017/S1368980026102031)
Supplement: Aktuna et al. supplementary material 2 — Aktuna et al. supplementary material [file S1368980026102031sup002.docx]

**Supplementary Table 2.** Numbers (n) and distribution (%) of the investigated population in which the socioeconomic determinants of obesity were evaluated according to sex, socioeconomic indicators and regional development

| **Regional development** | **High** | **Low** | **Total** |
| --- | --- | --- | --- |
|  | Unweighted, n / Weighted, n (%) | Unweighted, n / Weighted, n (%) | Unweighted, n / Weighted, n (%) |
| **Men** |  |  |  |
| **Education** |  |  |  |
| <8 years | 2,111 / 5,951,145 (33.1) | 1,433 / 4,213,737 (40.2) | 3,544 / 10,164,882 (35.7) |
| ≥8 years | 4,175 / 12,001,549 (66.9) | 2,083 / 6,276,555 (59.8) | 6,258 / 18,278,104 (64.3) |
| **Income** |  |  |  |
| Highest | 1,958 / 5,425,189 (30.2) | 565 / 1,542,997 (14.7) | 2,523 / 6,968,186 (24.5) |
| Second highest | 1,554 / 4,543,386 (25.3) | 567 / 1,650,714 (15.7) | 2,121 / 6,194,100 (21.8) |
| Middle | 1,345 / 3,763,425 (21.0) | 672 / 1,897,715 (18.1) | 2,017 / 5,661,140 (19.9) |
| Second lowest | 1,013 / 2,819,088 (15.7) | 778 / 2,201,421 (21.0) | 1,791 / 5,020,510 (17.7) |
| Lowest | 416 / 1,401,605 (7.8) | 934 / 3,197,445 (30.5) | 1,350 / 4,599,051 (16.2) |
|  |  |  |  |
| **Women** |  |  |  |
| **Education** |  |  |  |
| <8 years | 3,111 / 8,546,829 (47.8) | 2,290 / 6,456,158 (59.5) | 5,401 / 15,002,987 (52.3) |
| ≥8 years | 3,502 / 9,321,142 (52.2) | 1,561 / 4,388,907 (40.5) | 5,063 / 13,710,049 (47.7) |
| **Income** |  |  |  |
| Highest | 1,880 / 4,806,862 (26.9) | 547 / 1,378,872 (12.7) | 2,427 / 6,185,734 (21.5) |
| Second highest | 1,577 / 4,302,674 (24.1) | 621 / 1,717,359 (15.8) | 2,198 / 6,020,033 (21.0) |
| Middle | 1,534 / 4,166,346 (23.3) | 791 / 2,158,784 (19.9) | 2,325 / 6,325,130 (22.0) |
| Second lowest | 1,104 / 2,973,371 (16.6) | 869 / 2,330,656 (21.5) | 1,973 / 5,304,027 (18.5) |
| Lowest | 518 / 1,618,718 (9.1) | 1,023 / 3,259,393 (30.1) | 1,541 / 4,878,112 (17.0) |
